# New Appliances and Things Medical

**Published:** 1900-01-13

**Authors:** 


### NEW APPLIANCES AND THINGS MEDICAL.

### THE HANDY LIGHT STRETCHER.

(FRED. FISH AND SON, SUFFOLK HOUSE, IPSWICH.)

THIS is an admirably light and handy stretcher, which has been designed by Miss Laird, of Woking. The frame, which is of bamboo, is of the utmost simplicity, consisting of two poles and two cross-bars, each running in wide hems at the sides and ends of the strong ticking of which the body of the stretcher is made. By removing the cross-bars from their position the whole affair can be rolled up without removing the long poles, so that it may be ready for use at a moment's notice; but, if preferred, the whole of the canvas part can be removed from the poles and kept in a separate bag. One great advantage of this stretcher seems to be that there is nothing in it to break or get out of order, and that the frame, being held in position by the ticking, if anything should go wrong short of actual fracture of the poles it could be rectified at once by anyone who could use a needle and thread. The specimen which we have seen with its light bamboo frame struck us as being a very convenient and useful form of stretcher to keep at hand in case of accident; but, perhaps, for use in institutions in which such an appliance would be in constant demand, the fittings might be made a little stronger. It is surprising, however, what a strain a good bamboo will stand.

